# Three orthogonal ultrasounds fabricate uniform ternary Al-Sn-Cu immiscible alloy

**DOI:** 10.1038/srep36718

**Published:** 2016-11-14

**Authors:** W. Zhai, B. J. Wang, H. M. Liu, L. Hu, B. Wei

**Affiliations:** 1 Department of Applied Physics, Northwestern Polytechnical University, Xi’an, 710072, China.

## Abstract

The production of Al based monotectic alloys with uniform microstructure is usually difficult due to the large density difference between the two immiscible liquid phases, which limits the application of such alloys. Here, we apply three orthogonal ultrasounds during the liquid phase separation process of ternary Al_71.9_Sn_20.4_Cu_7.7_ immiscible alloy. A uniform microstructure consisting of fine secondary (Sn) phase dispersed on Al-rich matrix is fabricated in the whole alloy sample with a large size of 30 × 30 × 100 mm. The numerical calculation results indicate that the coupled effect of three ultrasounds promotes the sound pressure level and consequently enlarges the cavitation zone within the alloy melt. The strong shockwaves produced by cavitation prevent the (Sn) droplets from coalescence, and keep them suspended in the parent Al-rich liquid phase. This accounts for the formation of homogeneous composite structures. Thus the introduction of three orthogonal ultrasounds is an effective way to suppress the macrosegregation caused by liquid phase separation and produce bulk immiscible alloys with uniform structures.

Aluminium based immiscible alloys, such as Al-In^1^ Al-Si-Pb^2^ and Al-Cu-Sn^3^ are good candidates for advanced bearings in automotive applications if the soft and ductile secondary (In), (Pb) and (Sn) phases are homogeneously distributed on the hard and strong Al, Al-Si and Al-Cu matrix. However, macrosegregation is the major problem during the solidification of such alloys^4^,^5^,^6^ due to the large density difference between the two immiscible liquid phases. The formation of heavily segregated or even layered structure not only deteriorates the wear resistance but also degrades other mechanical properties, which limits their wide application. Therefore, great efforts have been made to explore novel methods to produce the desired uniform composite structure. In recent years, liquid state methods, such as rapid solidification^7^ and melt spinning^8^, semi-solid state technologies such as rheo-diecasting^2^, and solid state methods like mechanical alloying^9^ and severe plastic deformation^10^,^11^ are well developed to process immiscible alloys. Nevertheless, the formation of homogenous composite structure for bulk immiscible alloys remains to be a great challenge.

It is well known that applying external physical fields is an effective way to control alloy solidification process^12^,^13^,^14^,^15^, among which the power ultrasound is proved to refine the grain size and promote the mechanical properties^16^,^17^,^18^,^19^,^20^. The most popular way for introducing ultrasound is to insert a vibrating ultrasonic horn into the solidifying liquid alloys. However, the disadvantage of this one dimensional ultrasound method reported by many investigators^21^,^22^,^23^ is that the ultrasonic effectiveness is always confined to a limited volume, beyond which weak or even no influence on the microstructures takes place. For example, our previous work^21^,^22^ demonstrates that by one dimensional 20 kHz and 500 W ultrasound, the formation of uniform structure can be only realized in about 20 mm length along the direction of sound wave propagation in ternary Al-Sn-Cu immiscible alloys. This mainly arises from the sharp attenuation of ultrasound intensity with distance in a solidifying alloy melt.

To overcome the disadvantage of one dimensional ultrasound, we have recently proposed by numerical simulation that the employment of three orthogonal ultrasounds can greatly enhance the sound pressure level and enlarge the cavitation volume^24^, which may strength the effect of ultrasounds on the resultant microstructure. In this work, three dimensional (3D) ultrasounds are experimentally introduced into the phase transition process of ternary Al_71.9_Sn_20.4_Cu_7.7_ immiscible alloy. The results demonstrate that applying three orthogonal ultrasounds is really an effective way to prevent the macrosegregation caused by liquid phase separation and to produce bulk immiscible alloys with homogeneous composite structures.

## Results

As reported in ref. 3, under equilibrium condition, the phase transition of ternary Al_71.9_Sn_20.4_Cu_7.7_ alloy begins with the liquid separation L→L_1_(Al-rich) + L_2_(Sn-rich) at 828 K. Then primary solid (Al) phase precipitates from liquid phase L_1_ at 814 K. When the temperature drops to 801 K, four phase monotectic reaction L_1_→L_2_ + (Al) + θ(Al_2_Cu) occurs. Finally, the liquid L_2_ phase solidifies through ternary eutectic transformation L_2_→(Sn) + (Al) + θ(Al_2_Cu) at 501 K. The solidified sample is composed of solid solution (Al) and (Sn) phases, and intermetallic compound θ(Al_2_Cu).

**Figure 1:**
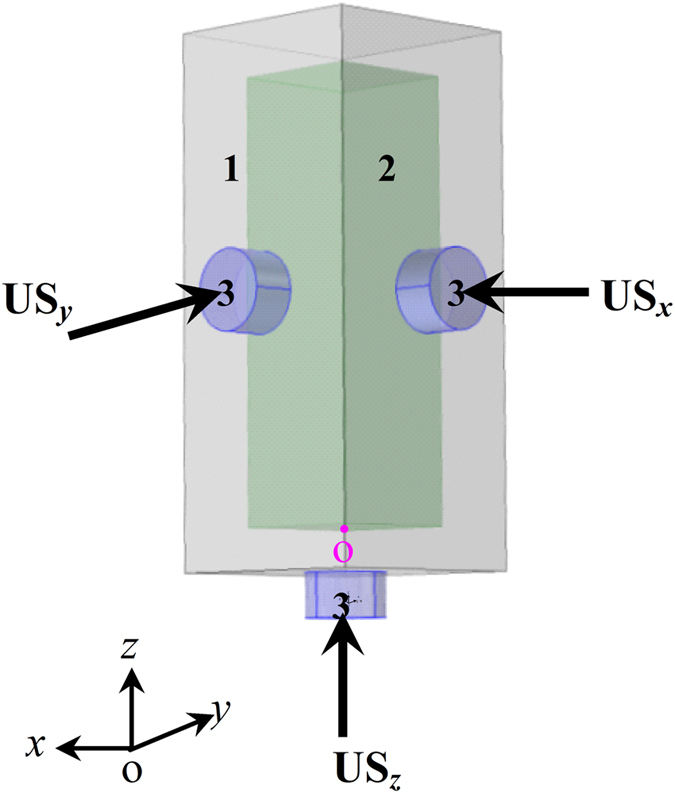
Schematic of experimental setup. 1, 2 and 3 denote casting mould, liquid alloy and three identical ultrasonic horns along *x*, *y* and *z* directions, respectively.

**Figure 2:**
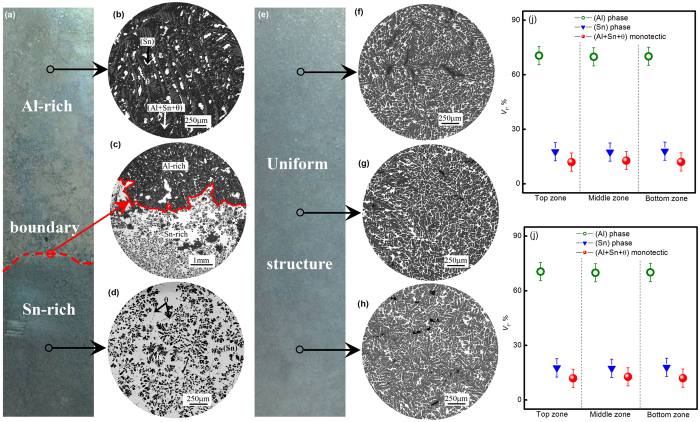
Solidification microstructure of ternary Al_71.9_Sn_20.4_Cu_7.7_ alloy: (**a**) layered-structure after static solidification; (**b**)~(**d**) structural morphologies in different locations during static solidification; (**e**) uniform structure under 3D ultrasounds; (**f~h**) microstructures of the top, middle and bottom parts of the alloy sample solidified under 3D ultrasounds. (**i**) volume fraction of different microstructures in Al-rich and Sn-rich zones in the static sample; (**j**) volume fraction of different microstructures in top, middle and bottom parts of the sample solidified under 3D ultrasounds.

As illustrated in Fig. 1, three orthogonal ultrasounds with the same frequency and vibration amplitude are introduced into the phase transition process of ternary Al_71.9_Sn_20.4_Cu_7.7_ alloy through the casting mould. Three dimensional coordinates *x*, *y* and *z* are set up, whose origin is placed at point O. The macro- and micro-morphologies of solidified alloy samples under static and 3D ultrasounds are shown in Fig. 2. As shown in Fig. 2(a), there is an evident boundary dividing the statically solidified sample into an upper Al-rich part and a bottom Sn-rich part, whose height fractions are about 58% and 42%, respectively. The enlarged views in the top Al-rich part presented in Fig. 2(b,c) are characterized by a large number of θ(Al_2_Cu) dendrites as well as small amounts of primary (Al) dendrites and secondary (Sn) phase distributed on the ternary (Al + Sn + θ) monotectic matrix. Statistical results on volume fraction (Fig. 2(i)) indicate that (Al + Sn + θ) monotectic structure occupies 62.20% volume of the top Al-rich part, and θ(Al_2_Cu) dendrites takes up 21.54% volume. By contrast, in the bottom Sn-rich part, as shown in Fig. 2(c,d), a few of (Al) and θ(Al_2_Cu) dendrites disperse on the (Sn) matrix, whose volume fractions are 23.42%, 8.36% and 68.22% (Fig. 2(i)), respectively. The morphological observation reveals serious macrosegregation in the statically solidified Al_71.9_Sn_20.4_Cu_7.7_ alloy sample.

**Figure 3:**
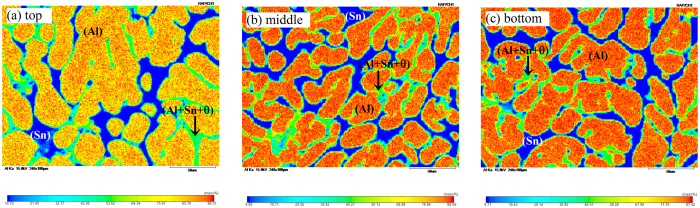
Uniform microstructures and solute distribution profiles of ternary Al_71.9_Sn_20.4_Cu_7.7_ alloy solidified within 3D ultrasounds analyzed by EPMA. A typical 240 × 180 μm area at (**a**) sample top; (**b**) middle part; (**c**) sample bottom.

Figure 2(e) shows the macroscopic pattern of Al_71.9_Sn_20.4_Cu_7.7_ alloy sample solidified within 3D ultrasounds. It is exciting that the whole sample seems homogeneous and no visible boundary can be found. As seen in Fig. 2(f)~(g), the solidification microstructures at sample top, middle and bottom parts are all featured by the uniform distribution of fine (Sn) phase dispersed on the Al-rich matrix. The corresponding volume fractions for (Al) dendrites, secondary (Sn) phase and ternary (Al + θ + Sn) monotectic structures are about 70%, 18% and 12% (Fig. 2(j)), regardless of their locations in the sample. Note that the accuracy of the volume fractions for different phases by conservative estimation is ±5%, and correspondingly, error bars of ±5% are applied for the volume fractions for different phases in both Fig. 2(i,j). Figure 3 displays the EPMA results on Al element distribution mapping by random choosing three areas with the same size (240 × 180 μm) at the top, middle and bottom parts of the alloy sample. The orange and red domains denote the high concentration of Al element, which is (Al) phase. The green zones denote moderate Al content, which correspond to the refined (Al + Sn + θ) monotectic structure, and can not be resolved at this magnification. By contrast, the blue regions with very low Al content are secondary (Sn) phase. Clearly, the similarities in growth morphology and solute distribution indicate the homogeneity in the whole alloy sample.

It also needs to be mentioned that we have previously found that when only one beam of ultrasound travels through the solidifying Al-Sn-Cu immiscible alloy, the uniformed structure can be produced only in the small region near the ultrasonic horn^21^,^22^. Here, it is encouraging that the application of three orthogonal ultrasounds results in the desirable homogeneous composite structure consisting of fine elongated (Sn) phase distributed uniformly on dendritic (Al) matrix, which provides a novel promising way in fabricating bulk uniform monotectic structure.

## Discussion

**Figure 4:**
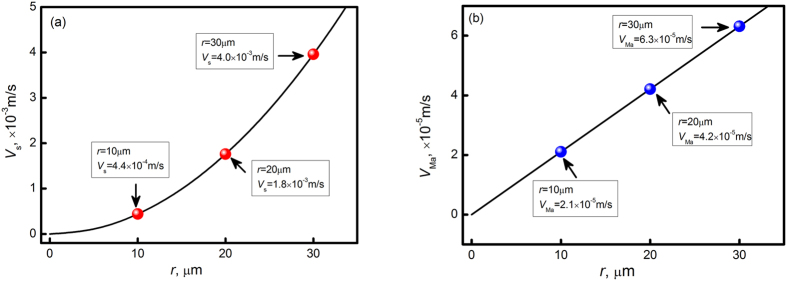
Stokes motion and Marangoni motion velocities of secondary (Sn) droplets: (**a**) Stokes motion; (**b**) Marangoni motion.

During the liquid separation process of ternary Al_71.9_Sn_20.4_Cu_7.7_ immiscible alloy under static condition, the secondary L_2_(Sn) droplets tend to sink down to the bottom of the sample by Stokes motion. Meanwhile, these tiny droplets also incline to move from the crucible wall to sample center, which is called Marangoni motion resulted from the transverse temperature gradient. These moving L_2_(Sn) droplets collide with each other to coagulate into large bumps, and then travel down to the sample bottom, which accounts for the formation of the layered structure shown in Fig. 2(a). The velocities for Stokes motion *V*_s_ and Marangoni motion *V*_M_ can be expressed as:


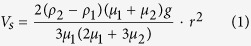



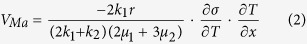


where *ρ*_1_ and *ρ*_2_ are the densities, *μ*_1_ and *μ*_2_ are the viscosities, and *k*_1_ and *k*_2_ are the thermal conductivities of L_1_(Al) and secondary L_2_(Sn) phase, *r* is the radius of L_2_(Sn) droplets, *g* is the gravity acceleration, ∂σ/∂*T* is the interfacial energy gradient, and ∂*T*/∂*x* is the transverse temperature gradient respectively. Figure 4 plots the calculated result on the velocities of both Stokes motion and Marangoni motion, and the physical parameters used in calculation are listed in Table 1. Assuming the L_2_(Sn) droplets with radius from 0 to 30 μm, the Stokes motion increases from 0 to 4.0 × 10^−3^m/s, whereas the Marangoni motion ranges from 0 to 6.3 × 10^−5^ m/s. Apparently, the Stokes motion is higher than the Marangoni motion by two orders of magnitudes, which plays the most important role on the formation of the layered structure.

It is well known that the cavitation effect is the major factor of ultrasound affecting the alloy solidification process. Here we calculate the acoustic field in one dimensional and three dimensional ultrasounds. The calculations have been performed in a casting mould with an inner size of 30 × 30 × 100 mm filled with homogeneous Al_71.9_Sn_20.4_Cu_7.7_ liquid alloy as shown in Fig. 1. The mould was made from steel with 10 mm in thickness. In one dimension ultrasound calculation, only an ultrasonic horn operated at 20 kHz is mounted at the bottom center areas of the mould, and it emits ultrasonic wave along *z* direction. In 3D ultrasounds case, as illustrated in Fig. 1, the three identical transducers are mounted at the center parts of the two lateral and bottom sides of the mould. They emit ultrasounds along three orthogonal directions.

The acoustic field within liquid Al_71.9_Sn_20.4_Cu_7.7_ alloy is numerically calculated on the basis of the modeling reported by refs 28, 29, 30, 31. The time harmonic wave propagation can be expressed by Helmholtz equation:


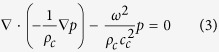


in which *p* is acoustic pressure, *ω* = 2π*f* is the angular frequency (*f* is the ultrasound frequency). The complex density *ρ*_c_ and sound speed *c*_c_ can be written as:


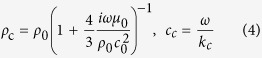


in which *k*_c_ is the complex wave number and *μ*_0_ is viscosity. *c*_0_ and *ρ*_0_ are the sound speed and the density of the media. The impedance boundary was utilized to specify the boundary condition of liquid alloy-air and steel mould-air interfaces. The impedance boundary is written as


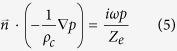


In equation (5), *Z*_e_ = *ρ*_e_*c*_e_ is the acoustic input impedance of the external domain, where *ρ*_e_ and *c*_e_ are the density and sound speed of the external domain. 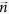 is unit normal vector against wall. For the transducer, the boundary was set as acoustic pressure. The initial pressure at the transducer were *p*_0_ = 0.94 MPa, corresponding to a vibration amplitude of 5 μm. The calculations were carried out by COMSOL Multiphysics™. In calculation, the casting mould and the inside liquid alloy were divided into 260,366 tetrahedron grids. The maximum and minimum lengths of the meshes are 2.65 and 0.50 mm, respectively. The physical parameters used in calculation are listed in Table 1.

**Figure 5:**
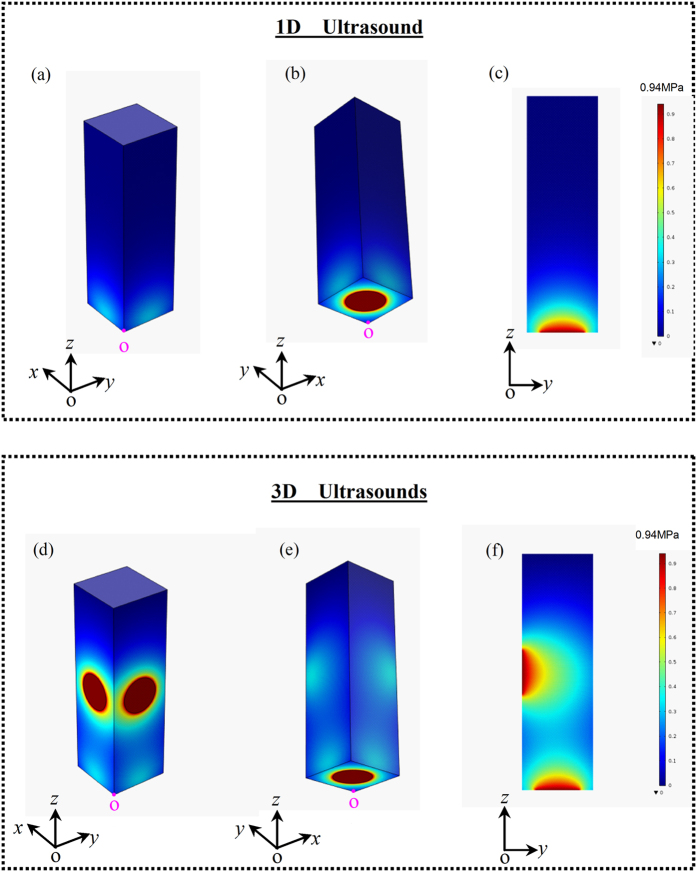
Numerical analysis on the sound pressure distribution within liquid Al_71.9_Sn_20.4_Cu_7.7_ alloy: (**a**)~(**c**) acoustic field in liquid alloy from different views within 1D ultrasound; (**d**)~(**f**) acoustic field in liquid alloy from different views within 3D ultrasounds.

**Figure 6:**
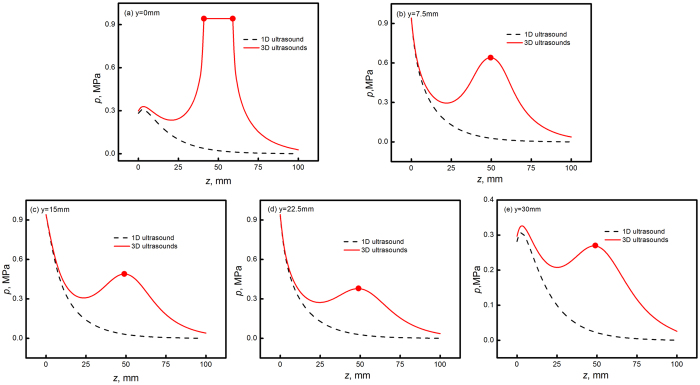
Comparison of sound pressure along different lines in the vertical section (x = 15 mm) of liquid alloy in 1D and 3D ultrasound cases: (**a**) y = 0 mm; (**b**) y = 7.5 mm; (**c**) y = 15 mm; (**d**) y = 22.5 mm and (**e**) y = 30 mm.

Figure 5(a)~(c) present the sound field distribution within liquid Al_71.9_Sn_20.4_Cu_7.7_ alloy in different views when 1D ultrasound propagates. It is apparent that the sound field exhibits symmetrical distribution along the *z* axis. The maximum sound pressure of 0.94 MPa occurs at the bottom of sample, which is close to the sound source. Then the sound pressure exponentially decreases along the wave propagation direction to the sample top. In a comparison, when 3D ultrasounds propagate, the sound field characteristics are shown in Fig. 5(d)~(f). The acoustic field appears maximum values at each sound source, though the maximum values keep at 0.94 MPa as the 1D ultrasound case. Figure 6(a)~(e) plot the sound pressure in different lines (*y* = 0, 7.5, 15, 22.5 and 30 mm) of the vertical section (*x* = 15 mm) along *z* axis. Clearly, the application of 3D ultrasounds strikingly promotes the sound pressure within the liquid alloy.

**Figure 7:**
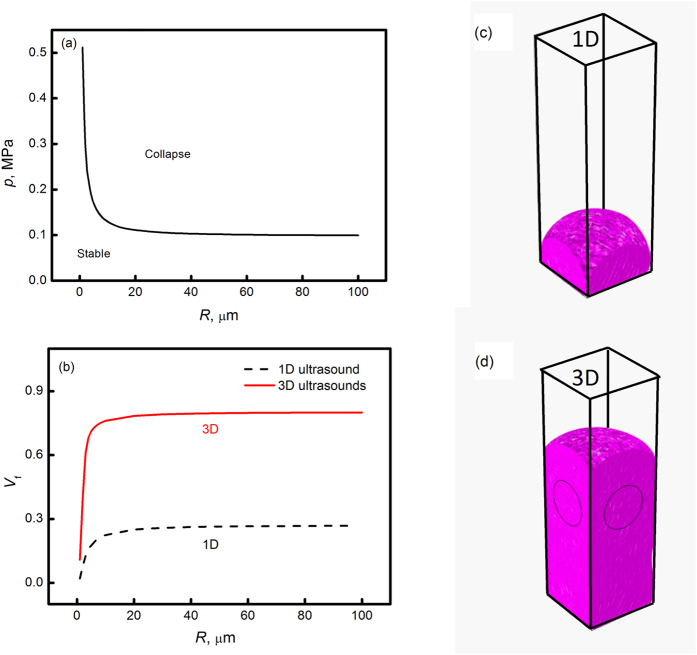
Calculated cavitation characteristics within liquid alloy: (**a**) sound pressure threshold for the collapse of bubbles with radius *R*; (**b**) the volume fraction for collapse of bubble versus radius; volume fraction for *R* = 10 μm bubbles collapse when induced by (**c**) 1D ultrasound and (**d**) 3D ultrasounds. The rose color in (**c,d**) represents for the cavitation zones in liquid alloy.

Furthermore, the potential cavitation zone induced by the acoustic field is calculated. The threshold sound pressure *p*_c_ for the collapse of bubble with radius *R* can be expressed as:


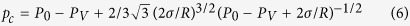


in which *P*_0_ is the static pressure, *P*_v_ is the saturated vapor pressure and *σ* is the coefficient of surface tension. Figure 7(a) presents the threshold sound pressure versus bubble radius. Assuming the bubble radius covers a span from 1 to 100 μm, the threshold sound pressure amplitude decreases from 0.5 to 0.1 MPa. Meanwhile, the corresponding volume fraction for the zones where bubble can collapse is illustrated in Fig. 7(b). It can be summarized that the volume fraction sharply rises with the increase of bubble radius from 1 to 20 μm. After that, it keeps at about 20% even if the bubble radius continuously increases. In contrast, if 3D ultrasounds are applied, the volume fraction for cavitation zones rises up to about 80%. The corresponding cavitation zone is marked in Fig. 7(c) under 1D ultrasound for such bubbles. It can be seen clearly that the cavitation can only take place within the sample bottom, which is close to the ultrasound source. As for the 3D ultrasounds, the cavitation volume drawn in Fig. 7(d) covers almost the alloy sample except the very top part. It also needs to be mentioned that here the discrepancy between calculation and experimental results mainly comes from the following two aspects. On one hand, we are not sure the size of the bubble existence in the liquid alloy, and the cavitation volume essentially depends on the bubble radius. In the calculation results shown in Fig. 7(c,d), we just showed that at a given bubble radius of 10 μm, about 20% and 80% volume for cavitation in liquid alloy induced by 1D and 3D ultrasounds, respectively. However, if there exist bubbles whose size is larger than 100 μm, the corresponding threshold sound pressure for cavitation will be reduced, and thus the cavitation can take place in even larger volume. On the other hand, in the calculation, the true values for many physical parameters, such as density, viscosity, surface tension and sound velocity of the liquid alloy etc. are not reported in any literatures. In this case, their values are linearly fitted by the corresponding values of the pure elements at their individual melting points. The uncertainty of these physical parameters used in calculation also results in the inconsistency between calculation and experimental results. Anyway, the common finding from the calculation and experimental results is that the application of 3D ultrasounds remarkably expands the cavitation volume in the liquid alloy as compared with that in 1D case, which prevents the macrosegreation of the two immiscible liquid phases.

The above results indicate that the enlarged cavitation volume induced by 3D ultrasounds takes the main responsibility for the formation of homogeneous composite microstructure within the whole alloy sample. During cavitation, the violent collapse of bubbles creates high-intensity shockwaves of the order of GPa magnitude and microjets of the order of 100 m/s in the alloy melt^31^,^32^. Note that this velocity is higher than the Stokes motion of L_2_(Sn) droplets. These intensive microjets are able to break up the L_2_(Sn) liquid droplets with large size and prevent them from coalescence, and can also encounter the Stokes motion by maintaining them suspended in the parent L_1_(Al) phase. By contrast, outside the cavitation zone, the very weak acoustic streaming has no impact on liquid phase separation^21^. This explains the reason why the uniform composite microstructure is only produced near the sound source when 1D ultrasound is introduced^21^,^22^ whereas formed within the whole sample once three orthogonal ultrasounds are applied in present work.

## Conclusions

The liquid phase separation and solidification process of bulk ternary Al_71.9_Sn_20.4_Cu_7.7_ immiscible alloy have been accomplished under 3D ultrasounds and compared with statically solidified alloy. In contrast to the layered structure formed due to macrosegregation under static condition, 3D ultrasounds lead to the desirable homogeneous composite structures consisting of fine elongated (Sn) phase distributed uniformly on dendritic (Al) matrix in the whole alloy sample with a large size of 30 × 30 × 100 mm. This is now qualitatively explained by the expanded volume of cavitation zones induced by the three orthogonal ultrasounds, which counteracts the Stokes motion of secondary (Sn) phase. This work proposes a promising way in fabricating uniform dispersion of secondary soft particles on hard matrix by adopting three orthogonal ultrasounds.

## Experimental Method

The experiments were performed in a solidification apparatus incorporated with three identical ultrasonic transducers. The ternary Al_71.9_Sn_20.4_Cu_7.7_ alloy samples were prepared from pure Al (99.99%), Sn (99.99%) and Cu (99.99%) elements, and melted by a resistance furnace. During experiment, the alloy melt with a superheating of 200 K was poured into a preheated rectangular casting mould with external dimension of 40 × 40 × 120 mm, and size of the alloy sample is about 30 × 30 × 100 mm. The ultrasonic horns are rigid coupling to the center areas of the two lateral and bottom walls of the casting mould along *x*, *y* and *z* directions, while the opposite walls were fixed immovably. Three orthogonal ultrasonic waves are introduced into the liquid alloys by these vibrating walls. The ultrasonic transducers were turned on until the liquid separation and solidification process finish. The vibration amplitude of the transducers was estimated to be 5 μm. After experiments, the solidified samples were vertically sectioned, mounted, polished and etched. The microstructure and solute distribution of solidified samples were analyzed by a vega 3 Tescan scanning electron microscope (SEM) and a Shimadzu 1720 electron probe microanalyser (EPMA).

## Additional Information

**How to cite this article**: Zhai, W. *et al.* Three orthogonal ultrasounds fabricate uniform ternary Al-Sn-Cu immiscible alloy. *Sci. Rep.* **6**, 36718; doi: 10.1038/srep36718 (2016).

**Publisher’s note:** Springer Nature remains neutral with regard to jurisdictional claims in published maps and institutional affiliations.

## Figures and Tables

**Table 1:** Physical parameters used in calculations.

Names and Units of the parameter	Value
Density of L_1_(Al) phase *ρ*_1_, Kg m^−3^	2385^25^
Density of L_2_(Sn) phase *ρ*_2_, Kg m^−3^	6980^25^
Viscosity of L_1_(Al) phase *μ*_1_, mN s m^−2^	0.1495^25^
Viscosity of L_2_(Sn) phase *μ*_2_, mN s m^−2^	0.5382^25^
Thermal conductivity of L_1_(Al) phase *k*_1_, W m^−1^·K^−1^	94.05^25^
Thermal conductivity of L_2_(Sn) phase *k*_2_, W m^−1^·K^−1^	30.0^25^
Interfacial energy gradient, ∂*σ*/∂*T*, J m^−2^ K^−1^	0.0003^26^
Transverse temperature gradient ∂*T*/∂*x*, K m^−1^	500
Ultrasound frequency *f*, KHz	20
Density of liquid alloy *ρ*_0_, Kg m^−3^	3759^25^
Viscosity of liquid alloy *μ*_0_, mN s m^−2^	0.24^25^
Sound velocity of liquid alloy *c*_0_, m s^−1^	3454^27^
Density of air *ρ*_e_, Kg m^−3^	1.2
Sound velocity of air *c*_e_, m s^−1^	343
Surface tension*σ*, N m^−1^	0.56^25^
Initial pressure at the ultrasonic horn *p*_0_, MPa	0.94

## References

[b1] Kaban I., Curiotto S., Chatain D. & Hoyer W. Surfaces, interfaces and phase transitions in Al-In monotectic alloys. Acta Mater. 58, 3406–3414 (2010).

[b2] Fang X. & Fan Z. Rheo-diecasting of Al–Si–Pb immiscible alloy. Scripta Mater. 54, 789–793 (2006).

[b3] Zhai W., Hu L., Geng D. L. & Wei B. Thermodynamic properties and microstructure evolution of ternary Al-10%Cu-x%Sn immiscible alloys. J. Alloys Compd. 627, 402–409 (2015).

[b4] Bertelli F. *et al.* Cooling thermal parameters, microstructure, segregation and hardness in directionally solidified Al-Sn-(Si, Cu) alloys. Mater. Design 72, 31–42 (2015).

[b5] Silva A., Spinelli J. E. & Garcia A. Thermal parameters and microstructure during transient directional solidification of a monotectic Al-Bi alloy. J. Alloy Compd. 475, 347–351 (2009).

[b6] Wang F. *et al.* Effect of solutal marangoni convection on motion, coarsening, and coalescence of droplets in a monotectic system. Phy. Rev. E 86, 066318 (2012). 10.1103/PhysRevE.86.066318 23368049

[b7] Nagase T. & Umakoshi Y. Microstructure of rapidly solidified Co-Cu-Si-B immiscible alloys with an amorphous phase. J. Alloy Compd. 650, 342–350 (2015).

[b8] Goswami R. & Chattopadhyay K. Melting of Bi nanoparticles embedded in a Zn matrix. Acta Mater. 52, 5503–5510 (2004).

[b9] Chithra S., Lele S. & Chattopadhyay K. Structure evolution and phase change in Ag–5.1 at.% Bi alloy during mechanical alloying. Acta Mater. 59, 2009–2019 (2011).

[b10] Wang M., Averback R. S., Bellon P. & Dillon S. Chemical mixing and self-organization of Nb precipitates in Cu during severe plastic deformation. Acta Mater. 62, 276–285 (2014).

[b11] Bachmaier A., Pfaff M., Stolpe M., Aboulfadl H. & Motz C. Phase separation of a supersaturated nanocrystalline Cu–Co alloy and its influence on thermal stability. Acta Mater. 96, 269–283 (2015).

[b12] Wang J. *et al.* Refinement and growth enhancement of Al_2_Cu phase during magnetic field assisting directional solidification of hypereutectic Al-Cu alloy. Sci. Rep. 6, 24585 (2016). 27091383 10.1038/srep24585 PMC4835779

[b13] Zhang L. M., Liu H. N. & Li N. The relevance of forced melt flow to grain refinement in pure aluminum under a low-frequency alternating current pulse. J. Mater. Sci. 31, 396–404 (2016).

[b14] Du L. F. & Zhang R. Phase-field simulation of concentration and temperature distribution during dendritic growth in a forced liquid metal flow. Metall. Mater. Trans. B. 45B, 2505–2515 (2014).

[b15] Jiang H. X., He J. & Zhao J. Z. Influence of electric current pulses on the solidification of Cu-Bi-Sn immiscible alloys. Sci. Rep. 5, 12680 (2015). 26228180 10.1038/srep12680 PMC4521156

[b16] Eskin G. I. & Eskin D. G. Ultrasonics treatment of light alloy metals. 2nd edn, 1–14 (London, 2005).

[b17] Tzanakis I., Lebon G. S. B., Eskin D. G. & Pericleous K. Investigation of the factors influencing cavitation intensity during ultrasonic treatment of molten aluminium. Mater. Design 90, 979–983 (2016).

[b18] Liu Z., Han Q., Huang Z., Xing J. & Gao Y. Ultrasound assisted salts-metal reaction for synthesizing TiB_2_ particles at low temperature. Chem. Eng. J. 263, 317–324 (2015).

[b19] Shu. D., Sun B. D., Mi J. W. & Grant P. S. A high-speed imaging and modeling study of dendrite fragmentation caused by ultrasonic cavitation. Metall. Mater. Trans. A 43, 3755–3766 (2012).

[b20] Chen R. R. A novel method for grain refinement and microstructure modification in Ti-Al alloy by ultrasonic vibration. Metall.Mater. Trans. A. 653, 23–26 (2016).

[b21] Zhai W., Liu H. M. & Wei B. Liquid phase separation and monotectic structure evolution of ternary Al_62.6_Sn_28.5_Cu_8.9_ immiscible alloy within ultrasonic field. Mater. Lett. 141, 221–224 (2015).

[b22] Zhai W., Liu H. M., Zuo P. F., Zhu X. N. & Wei B. Effect of power ultrasound on microstructural characteristics and mechanical properties of Al_81.3_Sn_12.3_Cu_6.4_monotectic alloy. Prog. Nat. Sci-Mater. 25, 471–477 (2015).

[b23] Qian M. & Ramirez A. An approach to assessing ultrasonic attenuation in molten magnesium alloys. J. Appl. Phys. 105, 013538 (2009).

[b24] Zhai W., Liu H. M., Hong Z. Y., Xie W. J. & Wei B. A numerical simulation of acoustic field within liquids subject to three orthogonal ultrasounds. Ultrason.Sonochem. 34, 130–135 (2007). 10.1016/j.ultsonch.2016.05.025 27773228

[b25] Gale W. F., Totemeier T. C. & Smithells C. J. Smithells Metals Reference Book 1102–1105 (Butterworth-Heinemann, 2004).

[b26] Wu M. H., Andreas L. & Lorenz R. Modelling the solidification of hypermonotectic alloys. Modelling Simul. Mater. Sci. Eng. 11, 755–769 (2003).

[b27] Iida T. & Gutthrie R. I. L. The Physical Properties of Liquid Metals 91–107 (Clarendon PressOxford, 1993).

[b28] Wu J. & Du G. Acoustic streaming generated by a focused gaussian beam and finite amplitude tonebursts. Ultrasound Med. Biol. 19, 167–176 (1993). 8516962 10.1016/0301-5629(93)90008-c

[b29] Nightingale K. R. & Trahey G. E. A finite element model for simulating acoustic streaming in cystic breast lesions with experimental validation. IEEE Trans. Ultrason. Ferr. 47, 201–215 (2000). 10.1109/58.818763 18238532

[b30] Xu Z., Yasuda K. & Koda S. Numerical simulation of liquid velocity distribution in a sonochemical reactor. Ultrason. Sonochem. 20, 452–459 (2013). 22634380 10.1016/j.ultsonch.2012.04.011

[b31] Eskin G. I. Broad prospects for commercial application of the ultrasonic (cavitation) melt treatment of light alloys. Ultrason. Sonochem. 8, 319–325 (2001). 11441617 10.1016/s1350-4177(00)00074-2

[b32] Komarov S. V., Kuwabara M. & Abramov O. V. High power ultrasonics in pyrometallurgy: current status and recent development. ISIJ Int. 45, 1765–1782 (2005).

